# Designing the Healthy Eating and Active Lifestyles for Diabetes (HEAL-D) self-management and support programme for UK African and Caribbean communities: a culturally tailored, complex intervention under-pinned by behaviour change theory

**DOI:** 10.1186/s12889-019-7411-z

**Published:** 2019-08-20

**Authors:** Amanda P. Moore, Carol A. Rivas, Stephanie Stanton-Fay, Seeromanie Harding, Louise M. Goff

**Affiliations:** 1 King’s College London, Departments of Diabetes & Nutritional Sciences, School of Life Course Sciences, Faculty of Life Sciences & Medicine, Room 3.87 Franklin-Wilkins Building, Stamford Street, London, SE1 9NH England; 2 Institute of Education, University College London, 18 Woburn Square, London, WC1H ONR England; 3 Department of Clinical, Educational and Health Psychology, Faculty of Brain Sciences, University College London, Alexandra House, 17–19 Queens Square, WC1N 3AZ London, England

**Keywords:** Type 2 diabetes, Black African and Caribbean, Behaviour change, COM-B, Participatory methods, Complex lifestyle intervention, Ethnicity

## Abstract

**Background:**

UK African and Caribbean (AfC) communities are disproportionately burdened by type 2 diabetes (T2D). Promoting healthy eating and physical activity through structured education is the cornerstone of T2D care, however cultural barriers may limit engagement in these communities. In addition, changes in lifestyle behaviour are shaped by normative influences within social groups and contextual factors need to be understood to facilitate healthful behaviour change. The Behaviour Change Wheel (BCW) and associated COM-B framework offer intervention designers a systematic approach to developing interventions. The aim of this study was to apply the BCW in the design of a culturally sensitive self-management support programme for T2D in UK AfC communities.

**Methods:**

An intervention development study was conducted. Focus groups were held with 41 AfC patients with T2D to understand healthful weight-management, diet and physical activity behaviours. The COM-B framework and BCW were used to evaluate the qualitative data, identify appropriate behaviour change techniques and specify the intervention components.

**Results:**

Participants were motivated to avoid diabetes-related consequences although did not always understand the negative impact of their current health behaviours on long-term diabetes outcomes. Barriers to healthful behaviour included gaps in knowledge related to diet, physical activity and weight management guidance. In addition, motivation and social opportunity barriers included an acceptance of larger body sizes, rejection of body mass index for weight guidance and cultural identity being strongly linked to consumption of traditional starches. There was a lack of social opportunity to perform moderate to vigorous physical activity, although walking and dance were culturally acceptable. The resulting Healthy Eating & Active Lifestyles for Diabetes (HEAL-D) intervention uses social support, social comparison, credible sources and demonstration as key behaviour change techniques.

**Conclusion:**

Use of COM-B and the BCW highlighted the need for an intervention to address motivational and social opportunity barriers to engaging in healthful behaviours, as well as addressing key gaps in knowledge. This framework facilitated the linkage of theoretical behaviour constructs with evidence-based behaviour change techniques, which will enable us to evaluate operationalisation of our chosen BCTs and their impact on behaviour change in a future feasibility study.

**Electronic supplementary material:**

The online version of this article (10.1186/s12889-019-7411-z) contains supplementary material, which is available to authorized users.

## Background

UK African and Caribbean (AfC) communities are disproportionately burdened with type 2 diabetes (T2D); prevalence is up to three times higher than in the general UK population [1] and it occurs at a younger age, with poorer control at diagnosis [2]. The high prevalence of T2D in UK AfC communities is attributed to a combination of genetic, socio-economic, lifestyle and cultural factors [3]. Promoting healthy eating, physical activity and weight loss through self-management education is the cornerstone of care for T2D [4]. However, cultural barriers, such as health literacy, lack of cultural salience of advice, and distrust of conventional medicine, may limit engagement of minority ethnic groups, including those of AfC ethnicity [2, 3]. Systematic reviews suggest that culturally tailored diabetes self-management education programmes are more effective than standard programmes [3, 5]. Culturally-tailored interventions for patients of AfC heritage have been developed and evaluated in US African American communities, however, transference to the UK is limited because there are many sociocultural differences. For example, African American communities experience financial barriers to healthcare while access to healthcare is free in the UK and African Americans have diets that are more assimilated to the general US population, than is evident for UK African and Caribbean communities [3, 6–8]. While the drivers of health behaviours in African American communities have been fairly well explored, there is a paucity of UK data to understand the attitudes and motivations that guide AfC patients to engage in positive health behaviours to support diabetes self-management.

Healthy Eating and Active Lifestyles for Diabetes (HEAL-D) is a programme of research in which a culturally tailored diabetes self-management and support programme has been developed and evaluated for UK African and Caribbean communities. Using UK Medical Research Council (MRC) complex intervention guidance, the intervention was developed using co-design methods to identify the theory by which it supports healthful behaviour change, before being evaluated in a feasibility trial. Drawing on the socio-ecological model (SEM) [9], the co-design work engages patients, community leaders and healthcare professionals to explore barriers and facilitators to diabetes self-management at the individual, community and healthcare system level. The Behaviour Change Wheel (BCW) [10] is an additional methodological framework that can aid intervention designers in understanding how to support behaviour change at an individual and intrapersonal level. It can provide a useful bridge between heuristic frameworks like the SEM and psychological behaviour change theory. The BCW helps intervention designers to understand influences on their target behaviours using the COM-B model, which conceptualises behaviour as being influenced by a person’s **capability** (their knowledge and skills), the **opportunity** to perform the behaviour afforded by the social and physical environment and a person’s **motivation** (their habits, feelings and thoughts). The BCW is then used to identify appropriate intervention functions and to link behaviour change techniques to the theoretical constructs [11–15].

The aim of this study was to apply the BCW and associated COM-B methodological framework to identify relevant behaviour change techniques (BCTs) and intervention components as part of the development of the HEAL-D culturally sensitive T2D self-management and support programme for people of AfC ethnicity.

## Methods

### Study design

An intervention development study was conducted using co-design methods. Data from patient focus groups were analysed using the BCW framework to identify appropriate behaviour change techniques and intervention components.

Ethical approval was granted in writing by King’s College London University Ethics Committee (LRS-15/16–3240) and the UK Health Research Authority (IRAS 194991). The study is reported according to COREQ guidelines (Additional file 4).

### Participants & setting

A purposive sample of adults of self-declared Black African (BA) or Caribbean (BC) ethnicity with a clinical diagnosis of T2D, living in London, were recruited face to face and by mail via GP surgeries in South London which have a high-density of AfC patients, face to face via local Black majority church networks and mosques, face to face through Diabetes UK Community Champions and via advertisements and researcher presence at local cultural events. A screening questionnaire was completed over the telephone with potential participants. A sampling framework was used to enable purposive sampling to achieve a broad representation of socio-economic circumstances (occupational class) and generational status (immigrant or born in UK). Effort was made to include groups not easily accessed and participants from both Christian and Muslim religions. For example, with the help of community leaders and advocates, Caribbean men were engaged via the church and focus groups were held within local mosques*.* During the screening telephone call, researchers explained that the research was to help inform the design of a new diabetes programme for AfC type 2 diabetes patients and what their involvement would entail. Exclusion criteria were the presence of chronic conditions that would significantly impact diet and activity behaviour, such as chronic renal conditions, and an inability to communicate in English. All participants were guided through an informed consent procedure by a member of the research team and gave their consent to participate, in writing.

### Qualitative procedures

Focus groups were conducted in community venues such as churches and mosques and in university premises. Focus group methodology was chosen, as our objective was intervention design, so we were interested in the convergent views of the communities taking part. As differences in health behaviour have been identified between genders and between ethnicities, groups were held separately with men and women and with participants of Caribbean and African ancestry [1, 8, 16]. Each group lasted approximately 2 h and was audio recorded. The topic guide was informed by a literature review and guidance for the use of the COM-B approach [10] and was loosely divided into three sections to cover diet, physical activity and weight (Additional file 3).

The intervention aimed to align with NICE guidance for structured education [4] and address key lifestyle targets from the UK evidence-based T2D diet and lifestyle guidelines [17]:

- Daily moderate to vigorous physical activity or 30 min, at least 5 days a week but every day if possible.
- Modest weight loss (5–10%) in overweight/obese and maintenance in those of healthy weight.
- Balanced carbohydrate intakes through portion control and consumption of low glycaemic index and wholegrain sources.
- Limited saturated and trans-fat intake, and replacement with mono-unsaturated fats, and oily fish consumption at least twice a week.
- Limit salt intake to less than 6 g per day.

The focus groups aimed to identify which of these guidelines were particularly challenging for AfC patients to adopt and which behaviours were potential targets for intervention.

Prompts such as photos of people of different body shapes and weights, together with examples of different foods, and video detail of the physical activity guidance for T2D, were used to promote free discussion and debate. The topic guide was modified iteratively through the research period to ensure optimal discussion of the key topics. The groups were conducted by two female authors (LG- PhD, Senior Lecturer and NIHR Research Fellow; AM – MSc, Doctoral Researcher) trained in qualitative techniques and with a history of research work in these communities, both of White-European ethnicity. The professional background of the researchers and the reasons for the study were introduced before the start of the session. One researcher facilitated the group while the other took detailed observational field notes of the sessions, switching roles between groups. Only the participants and researchers were present during the groups.

The focus group recordings were transcribed and the transcripts checked against the audio recordings for accuracy. An a priori framework approach was adopted to assist analysis of the data, with deductive codes based on COM-B and the associated Theoretical Domains Framework (TDF) (Table 2 & Additional file 1) [18]. NVivo software was used for data management. Twenty percent of the data was independently coded by two researchers and discrepancies discussed and recoded until consensus, to improve validity. Preliminary themes within the main COM-B categories were identified and the resulting data reviewed with a third author, acting as an independent peer reviewer. As the objective of this research was to inform cultural adaptations for a community diabetes support programme, only the commonly occurring convergent themes are included in the analysis. Data saturation was considered to have been achieved once no new themes were emerging. Once the key themes were agreed, the data were validated with a stakeholder advisory group made up of patients from the target population and community leaders. The group comprised 3 community advocates who were involved in recruitment and 7 patients who volunteered to form a patient advisory group for the length of the study. A group meeting was held to feedback key findings and to confirm the analysis resonated accurately within the target population.

### Analysis using the COM-B framework

Details of the use of the COM-B framework, as recommended by Michie et al. [10], were as follows:

i. *a) Defining the target behaviours & b) understanding what needs to change for the behaviour to occur*: the focus group data were used to develop an understanding of the influences on the health behaviours associated with following the guidelines and to prioritise the key behavioural targets pertinent and salient for these communities.
ii. *Defining intervention functions and choosing appropriate behaviour change techniques:* intervention functions were identified and appropriate BCTs selected from the Behaviour Change Taxonomy (v1) [15, 19]. The APEASE criteria of Acceptability, Practicability, Effectiveness, Affordability, Safety and Equity, were applied to the short-list of potential BCTs [10]. In addition, review level data for BCTs most commonly deployed in successful lifestyle interventions were used to assist in the selection of potential BCTs [20–22]
iii. *Specifying intervention components and intervention delivery:* intervention components were specified to support the chosen BCTs and decisions about the mode of delivery were made. All focus group participants and community advocates were invited to two co-design workshops and 27 attended. The workshops involved the use of photo prompts, break-out group discussion and presentation of group views, to gain feedback on the validity of the analysis and to jointly agree the intervention content.

## Results

Forty-one participants took part in 8 focus groups (Table 1) which were held in July–December 2016. Participants were primarily first generation and 44 % were educated to a basic level (16 or younger). All consented participants took part in the discussions.

**Table 1:** participant characteristics

Characteristics	Total sample *n* = 41	Black African *n* = 23	Black Caribbean *n* = 18
Age (SD)	62.4 (11.7)	59.5 (12.1)	66.1 (10.4)
% Female (*n*)	66 (27)	70 (16)	61 (11)
% Born outside UK (*n*)	88 (36)	91 (21)	83 (15)
Educational attainment^a^			
% Basic (*n*)	44 (18)	30 (7)	61 (11)
% Secondary (*n*)	27 (11)	30 (7)	22 (4)
% Tertiary (*n*)	24 (10)	35 (8)	11 (2)
Employment status^b^			
% Retired (*n*)	46 (19)	39 (9)	56 (10)
% Part-time^b^ (*n*)	17 (7)	22 (5)	11 (2)
% Full-time (*n*)	22 (9)	22 (5)	22 (4)
% unemployed (*n*)	10 (4)	13 (3)	6 (1)

^a b^ 2 participants did not provide educational or employment information

### i a) Defining target behaviours

We brainstormed a list of possible target behaviours based upon the UK evidence-based T2D diet and lifestyle guidelines [17]. Focus group data and existing literature were used to prioritise behavioural targets that were most likely to be helpful in these communities and behaviours were scored according to their potential impact, likelihood of being achieved, ease of measurement and potential spill-over to other behaviours (Additional file 2) [10]. For example cultural preferences were evident from the focus groups with regard to some of the potential behaviours; the qualitative data indicated that weighing food portions was unlikely to be effective *“we measure with our eyes*” (*BA male)* and the use of weight targets based on BMI was strongly rejected as being culturally inappropriate, *“They say that we’re obese because we’re heavy-boned and … because you’re measuring a black lady on a European chart. How are we obese?” (BC female).*

Four key behaviour targets were prioritised:

- Reduce carbohydrate portion size at each meal to a fist or palm size (equivalent to 50 g carbohydrate).
- Switch saturated fat sources to unsaturated fats.
- Do 30 min moderate to vigorous physical activity at least 5 days a week.
- Monitor waist size to achieve/maintain it below recommended targets (80 cm for women and 94 cm for men).

### i b) Understanding influences upon the behaviour(s)

A summary of the COM-B behaviour analysis for each selected behaviour is given in Table 2 and described in detail below:

#### Behaviour 1: To reduce carbohydrate portion size at each meal to a fist or palm size

**Table 2:** COM-B coding & analysis of selected behaviours

COM-B Domain		Coding included in this domain	What needs to happen for the behaviour to occur?			
			Reducing carbohydrate to palm/fist each meal	Switch saturated fats for unsaturated fats	Do 30 mins MVPA 5 days a week	Monitor waist circumference
Capability	Psychological	Behavioural regulation Cognitive skills Interpersonal skills Knowledge Memory Decision making processes	Knowledge: Understand which foods are carbs; understand portion size targets and what foods can replace carbs to avoid hunger. Behavioural regulation & cognitive skills: To set goals and maintain motivation. Have strategies for eating socially.	Knowledge: Know what foods contain different oils. Learn to cook differently Behavioural regulation & cognitive skills: To set goals and maintain motivation. Have strategies for eating socially.	Knowledge: Know what the guideline targets are. Know what activities are suitable.	Knowledge: Know what the guideline targets are.
	Physical	Physical skills			Have stamina and skill to undertake activity	Know how to accurately take the measurement
Opportunity	Social	Social influences	Have social support to change behaviour.	Have social support to change behaviour.	Have social support to change behaviour.	Have social support to change behaviour.
	Physical	Environmental context Resources and equipment			Have suitable activities accessible & affordable. Have the time to undertake the activity.	Have a tape measure.
Motivation	Reflective	Social role & identity Beliefs about one’s own capability Optimism Beliefs Intentions Goals	Identity: Address issues of cultural identity portion size. Beliefs about consequences: Understand the mechanisms of glucose balance and the consequences of not achieving targets.	Identity: Address issues of cultural identity associated with cooking in oil & food choice. Beliefs about consequences: Understand the consequences/benefits to change and know the risks of saturated fats.	Self-belief: over-come fears of injury. Identity: Address issues of cultural identity associated with exercise. Beliefs about consequences: Understand why achieving the guidelines is important.	Beliefs: overcoming acceptance of larger body sizes. Beliefs about consequences: Understand why achieving the guidelines is important.
	Automatic	Emotion Reinforcement/habit	Overcome hunger and cravings Support habit formation with planning skills	Get used to new tastes & flavours Support habit formation with planning skills	Support habit formation with planning skills	Support habit formation with planning skills

##### Capability (knowledge & skills)

General knowledge of the key dietary principles for diabetes management was good, for example the need to increase vegetable intake, cut down on saturated fat, increase fibre and reduce intake of fried foods. However, there were gaps in knowledge and confusion around what foods contributed to blood glucose fluctuation; participants did not always understand that natural sugars and added sugars were carbohydrate and similarly starches such as rice, potatoes and other ‘hard foods’ such as yam and green banana, were not always understood to contribute to elevate blood glucose.

*“I use a lot of smoothies and I was told that it goes straight into your bloodstream, so I’m basically really confused as to what I can have and what I shouldn’t have and the reason why I shouldn’t have it.”. BC Male, Focus group 4.*

The majority, but not all, of participants had received dietary advice from healthcare professionals. However, participants reflected that the advice couldn’t be translated to the foods they usually ate. Portion advice wasn’t always easy to understand because many staple foods, for example maize meal or gari, are made to different consistencies, making it difficult to judge how much carbohydrate is in a portion.

*“When I went to the course [diabetes education course] I was telling them about breadfruit, they didn’t know what I was talking about. They didn’t really give us anything on West Indian food…they just showed pictures of fish, bread and potatoes … when I went home I was thinking, well, what levels of sugar are there in yam?”. BC Female, Focus group 8.*

*“some want it strong … some soft … .so when you try to discourage eating a [large] portion, sometimes it doesn’t work”. BA Male, Focus group 5.*

As a result of participant discussion, we understood that managing food intake required problem-solving skills, will power and understanding about the role different foods play in diabetes management.

*“It’s a lot of hard work being diabetic because you have to consciously think about what you’re putting in your body … it’s every day you have to think consciously about what you’re eating”. BA Female, Focus group 6.*

##### Opportunity

Social opportunity to reduce portion sizes was limited by social norms to eat large portions, the central role food plays in the social fabric of the community, and the role fruit and sugar play in the traditional diet.

*“We tend to have solid portions. And them the portions that we are used to, you have to eat and have your belly full”. BA Female, Focus group 3.*

*“Everything’s food, so in big gatherings and funerals and stuff – it’s food. If you’re not talking about it, you’re thinking about it”. BC Female, Focus group 8.*

*“I’ve got a mango tree at home and I go to Jamaica every year especially for that and I sit there and eat for a couple of days”. BC Female, Focus group 8.*

However, support from family members motivated healthful dietary behaviour both in the home and throughout everyday life.

*“My children, they go, ‘Mummy, you need to watch your diet. You need to do this’”. BA female, Focus group 3.*

*“My sisters and I … .we do a lot of talking about what are you eating, what you are doing, taking pictures of food, sometimes good, sometimes bad …*. *So we do it a lot. So the support helps when you’re ready to do it”. BA Female, Focus group 6.*

Men were largely reliant on the food provided by their wife or partner, which sometimes resulted in eating larger portions, if that was what was served, possibly because the partner did not understand or support the diabetes-related dietary recommendations.

*“Just my wife and she already decides things that I eat, decides things that I drink”. BA Male, Focus group 5.*

##### Motivation

Motivation to reduce carbohydrate portion sizes was limited by the fact that traditional carbohydrates form the essence of the traditional diet. Advice to reduce carbohydrate portion sizes was understood as being told not to eat traditional foods, particularly amongst the African participants and consequently they were unlikely to follow advice.

*“It’s hard, it’s very hard, because you are a person who usually is maybe - eat rice in the morning, then maybe rice again in the afternoon, then maybe amala in the evening. Then, all of a sudden, you have to go without all this food because it’s something you’ve been brought up”. BA Female, Focus group 3.*

*“I eat mostly our food and I enjoy it, I can’t leave it, no matter what the doctor says”. BA male, Focus group 5.*

The consequences of cutting down carbohydrates could be seen as both positive and negative. It was associated with hunger in all groups. However, participants still recognised that they needed to make changes if they were to avoid future complications.

*“He told what I cannot eat but if I followed the doctor’s orders I will be starving”. BA Male, Focus group 5.*

*“You want to live longer and if you don’t control yourself … ..at the end of the day you have to help yourself”. BC Male, Focus group 2*

*“We can see the complication, there are some people with amputated legs, some people with blindness”. BA Female, Focus group 3.*

#### Behaviour 2: Switch saturated fat sources to unsaturated fats

##### Capability

Knowledge of the benefits of low-fat diets for health was generally good, however, participants did not always understand the differences between saturated and beneficial unsaturated fats and food sources of these. For example, one participant described how people were avoiding eating beneficial fish oils because they thought they needed to cut down all fat.

*“They were saying that mackerel fish …*. *they don’t normally eat it anymore … in Nigeria they were telling me that their doctors, their nurses said that they shouldn’t … I said, ‘Why?’ She said, ‘They said it’s too oily.’”. BA female, Focus group 3.*

##### Motivation

The main barrier to cutting down on saturated fat and increasing unsaturated fat was that oil was believed to be central to the traditional diet and foods that were traditionally cooked with oil were more difficult to enjoy if cooked in a different way.

*“We is proper West Indian we season we fry”. BC Female, Focus Group 8.*

*“Even when you’re eating your soup you want that [palm oil] on top”. BA Female, Focus group 3.*

#### Behaviour 3: Do 30 min moderate to vigorous physical activity at least 5 days a week

##### Capability

There were both psychological and physical capability barriers to engagement in moderate to vigorous physical activity. There was confusion as to what activity they needed to do and how much, and they perceived healthcare practitioners’ guidance to be vague. In addition, those with injuries did not have the knowledge, skills or stamina to try alternative exercises such as chair-based routines and so did not engage in exercise.

*“There are no real tools … you’re being told you need to exercise … … what does that mean?”. BA Female, Focus group 3.*

*“I can’t do these things because I can’t stand for a long time”. BA Female, Focus group 3.*

##### Opportunity

Women reported that purposeful vigorous exercise was not the social norm *“Going to the gym is not in my culture. It’s not in my culture at all”. BA Female, Focus group 6.* This limited the social opportunity to engagement. However, there was a strong cultural association with dancing and walking as part of everyday life.

*“So the people sing and dance and clap so even the old people they dance a lot”. BC Male, Focus group 2.*

*“We’re used to walking we walk a lot”. BA Female, Focus group 6.*

This meant that the daily use of public transport, particularly the bus network, presented an opportunity to build more walking into daily activity. The social interaction of walking with friends and group activities also encouraged exercise participation and friends and family supported engagement in physical activity.

*“It helps to walk and talk … you don’t think of the journey … you just walk and you know, talk and you feel it nice”. BC Female, Focus group 1.*

*“I think it’s the camaraderie of a group … you get to know people. Support … when you walk in a group you can hear more from people what they are saying and how they treat themselves”. BA Female, Focus group 3.*

For those who did have intention to exercise, barriers included lack of time and tiredness as many participants reported having multiple jobs and doing shift work as well as juggling caring roles within the family. In addition, cost of classes or gym membership were seen as prohibitive, although this could be overcome where GP offered free exercise referrals.

*“The exercise issue, this country is a very busy country, I’m working, sometimes I just become a bit lazy, a couple of days, sometimes two weeks, I don’t really have time because I come back from work, I’m tired. … go to bed”. BA male, Focus group 5.*

*“It’s on your library card, so then you get free use … with diabetes, to keep sugars corrected and it has to be a medical referral for it to be free”. BC Female, Focus group 8.*

##### Motivation

Women described themselves as having active lives, for example, taking public transport, walking and caring for grandchildren. However, motivation to engage in moderate/vigorous physical activity was limited by participants’ beliefs that they were active enough and avoiding sedentariness was sufficient. While participants knew that being active was beneficial for T2D, very few knew of the direct relationship between activity and blood glucose levels. They also feared injury.

*“I do a lot of exercise in my domestic time …I don’t sit one minute. So, that’s my exercise really and I don’t think I will take up fully exercise. I think I do enough domestic one”. BC Female, Focus group 1.*

*“I went on one of them [exercise bike] and my knee seems to have come lose or something. I had to be up on walking sticks”. BA Female, Focus group 7.*

In addition, there was a belief amongst men that once you achieve fitness you can reduce the amount of moderate/vigorous activity you need to do, rather than recognising the need to be active most days if you have T2D.

*“I’m fit so I’m alright … I spend a good time – like once a week … so I’m alright”. BC Male, Focus group 4.*

Conversely however, positive emotional benefits and camaraderie motivated engagement in walking.

*“The walk is the big one. I love walking”. BA Female, Focus group 7.*

*“It’s the open space … the clearing of the mind … .that’s what I get out of it … It’s awesome and being in the fresh air”. BC Female, Focus group 8.*

#### Behaviour 4: Monitor waist size to meet recommended cut offs

##### Capability

Participants had a good knowledge of the health risks of *‘middle fat’ (BA Female),* particularly for diabetes, although very few knew the actual targets for waist size.

*“I think she’s higher risk … .because they measure by the waist”. BA female, Focus group 7.*

##### Opportunity

Social norms supporting a desire for a smaller waist were evident, for example women thought it important to fill out the shape of traditional dresses and to be curvaceous.

*“She doesn’t look big because she fits what she’s got on [curves of the traditional dress]”. BC female, Focus group 8.*

*“The skinny-skinny ones, they [Men] don’t want no [skinny] person; they want a woman they can feel, a voluptuous woman …*. *so the guys will go for the bigger ones, so yes, they’ll get married first”. BC Female, Focus group 8.*

##### Motivation

Motivation for having a smaller waist whilst retaining a curvy shape, resonated with cultural values to a greater extent than overall desire for weight loss to be ‘skinny’. Weight around the abdomen was seen as a trigger that weight reduction was needed.

*“I don’t want to lose weight and change but I’m trying to alter my shape, like my trousers. I used to wear 42 but now I wear 38”. BA male, Focus group 5.*

*“I never try to lose weight but yeah, if my tummy higher …I stop eat grease”. BA female, Focus group 7.*

### ii Identification of intervention functions and choice of behaviour change techniques

**Fig. 1:**
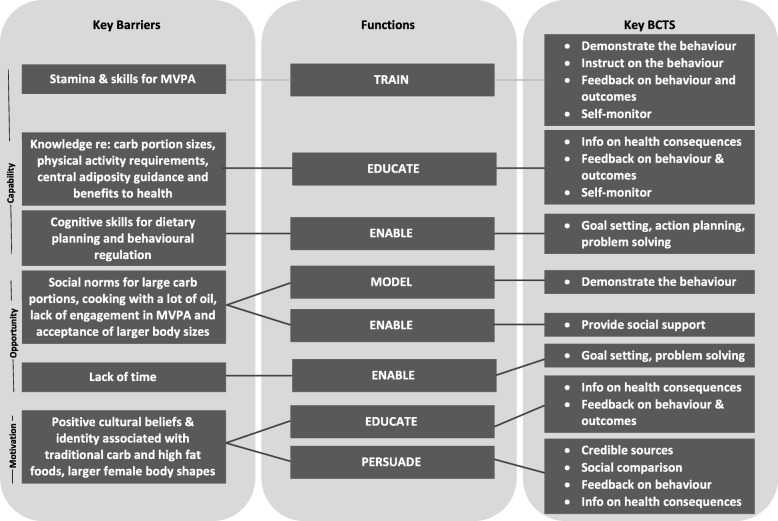
Overview schematic to show the key BCTs used in the HEAL-D programme and how they link to the main barriers to healthful dietary and physical activity behaviour

The mapping of the qualitative data to each of the sub-categories of the COM-B domains identified that the intervention needed to perform the functions of education, persuasion, training, modelling and enablement (Fig. 1).

Using the APEASE approach, BCTs were chosen to develop capability (knowledge and skills) around food choice and exercising; improving motivation to reduce portion sizes, cook with less saturated fat, and monitor waist size; and develop social opportunity to achieve all four of the target behaviours (Fig. 1 and Table 3). The BCW suggests possible BCTs for each intervention function identified. We made a pragmatic short-list of BCTs from the suggestions, based on the research team’s knowledge of the community, the focus group data and systematic review data of commonly used BCTs in successful lifestyle interventions. These were then scored according to the APEASE criteria. For example, biofeedback (such as use of a glucose monitor) was a possible BCT identified from the BCW and the focus group data, to educate and persuade, however, it was not considered affordable in the context of the intervention. Providing social support and social comparison and the use of credible sources and demonstration were identified as particular key BCTS for these communities as they were commonly cited as helpful during the focus group discussions.

**Table 3:** Intervention functions and chosen behaviour change techniques

Target Behaviour	Behaviour change techniques											
	Social support (unspecified)	Social comparison	Credible sources	Information about health consequences	Feedback on outcomes	Self-monitoring of behaviour	Instruction	Demonstration	Graded tasks	Goal setting (behaviour)	Problem solving	Action planning
Reduce carbohydrate portion size at each meal to a fist or palm size	□	□	□	□	□		□	□		□	□	□
Switch saturated fats to unsaturated fats	□	□		□	□		□	□			□	□
Do 30 min moderate to vigorous physical activity at least 5 days a week	□	□	□	□	□	□	□	□	□	□	□	□
Monitor waist size to meet recommended cut offs	□	□		□		□	□				□	□
Intervention function	Persuasion Enablement	Persuasion	Persuasion	Education Persuasion	Education Persuasion Training	Education Training	Training	Training	Training	Enablement	Enablement	Enablement

### iii Development of the intervention strategies and components

Clinical guidelines recommend group-based delivery of self-management education sessions [4] and our analysis identified benefits of group interaction and support in this population supporting the appropriateness of delivering HEAL-D in face to face groups. It will be delivered by a dietitian and lay educator in 7 community-based sessions.

The development of the intervention components was influenced by the focus group data and the resulting choice of BCTs (Table 4). **Information about health consequences** and the benefits of the target behaviours, particularly portion control of traditional carbohydrates and the need for regular brisk exercise, was incorporated into an education curriculum covering the principles of self-management. To provide the **social support** and **social comparison** the participants desired, sessions were designed to stimulate group discussion and sharing of successes and challenges around specific group tasks undertaken during and between sessions. In addition, communication between participants was encouraged and facilitated by the use of social media. **Demonstration, training** and **instruction on how to perform behaviours** were delivered by including practical cooking sessions, physical activity classes delivered by trained instructors and hands-on games and activities to demonstrate food/meal composition. **Graded** [exercise] **tasks** were used to increase self-efficacy to exercise and to overcome injury fears and mobility restrictions that participants reported. **Credible sources** and **social comparison** techniques informed the development of videos featuring patients and healthcare professionals talking about self-management, which were designed to persuade and increase motivation to change. **Goal setting** and **feedback on outcomes of behaviour** were built into the curriculum with the provision of the patients’ own measurements and blood results, to help improve motivation to overcome reported barriers. In addition, **self- monitoring of behaviour** was encouraged through provision of pedometers and behaviour diaries. The curriculum also aims to develop **goal setting** and **action planning** skills using participant generated examples and SMART goals (Specific, Measurable, Achievable, Realistic, Time-based) [23].

**Table 4:** Description of the intervention components to support each behavioural change technique

BCT	Intervention component
Social support (unspecified)	Social connectedness will be fostered within the group by the discursive nature of the sessions and through shared engagement in activities and structured exercise sessions
Social comparison	The *‘homework’* activities will give participants opportunity to try the lifestyle targets and come back to discuss with the group and with educators. Participants will be encouraged to share their successes to encourage comparison within the group. In addition, role models will be featured in the case study video
Credible sources	Videos will be used as part of the intervention which include advice and tips from community leaders, healthcare practitioners and patients from the community that have successfully changed their habits
Information about health consequences	The educational curriculum will cover health consequences and benefits of various key lifestyle behaviours A video will explain the mechanisms of type 2 diabetes
Feedback on outcomes, self-monitoring of behaviour	Programme will start with personal measurements and blood results, and updated outcome measures will be given at the end of the programme. They will be encouraged to monitor their waist measurements through the course by completing their programme booklets.
Self-monitoring of behaviour, action planning	Participants will be given pedometers to measure their steps and will be taught to develop action plans and measure their progress against them.
Instruction on how to perform the behaviour	The curriculum will communicate health guidance clearly using culturally relevant examples.
Demonstration	Practical games, the weekly discussion tasks, a cooking session and structured exercise sessions will provide guided demonstration. An exercise DVD will be provided for participants to follow at home.
Graded tasks	Physical activity sessions and targets will be graded for ability to boost chances of success hence confidence and self-efficacy.
Goal setting (behaviour)	Participants will be guided through setting their own goals for the lifestyle targets that are important for them
Problem solving	The *‘homework’* activities will be discussed at the beginning of each session, challenges will be identified and the group will problem solve collectively. Problem solving will also form part of the education sessions about lifestyle habits.
Action planning	Participants will be guided through how to develop and adjust action plans for each of the target behaviours and for their personal objectives, to help keep them motivated.

## Discussion

We used the COM-B framework and the BCW to inform the design of a theoretically underpinned culturally sensitive diabetes education and support programme for AfC patients with T2D. Importantly we have identified that motivation and opportunity to perform healthful diabetes-related self-management behaviours in AfC patients may be limited by specific cultural beliefs and cultural social norms, even in the presence of adequate levels of knowledge. Our data have highlighted powerful cultural barriers to the use of BMI and weight targets as part of the healthcare interaction and the identification of waist circumference as an acceptable alternative that aligns more closely with cultural beliefs. Our behavioural analysis identified that our intervention should focus on the functions of education, training, enablement, modelling and persuasion. The most impactful BCTs to support these functions were considered to be providing social support, social comparison and information from credible sources, including members of the community, to help shape beliefs about positive diabetes-related health behaviours and provide social support to adopt and motivate change. In addition, self-monitoring of behaviour and outcomes and action planning were also judged to be important to support behavioural regulation, increase self-efficacy and motivation and to help the formation of positive habits.

The BCW framework provides a relatively simple, accessible methodology for use by intervention designers. It helps describe the theory of an intervention and identify proposed mechanisms of behaviour change, in order to improve the likelihood of effect from an intervention, as well as allowing the explicit description of BCTs for data synthesis between studies [24]. Many examples of the use of BCW in the literature describe relatively straightforward interventions in homogenous populations, whereas our intervention needed to target multiple behaviours, with complex influences, in a fairly diverse population in terms of their age, degree of acculturation and current lifestyle habits. As a result, we limited our selection of target behaviours and specified them less precisely than is suggested in the BCW. In this way we were able to develop a programme that worked amongst our diverse target population e.g. with a goal to do 30 mins of moderate to vigorous activity at least 5 days a week, rather than specifying exactly what activity should be done and where and when it should be done. We then introduced classes demonstrating a range of activities into the intervention based on the data, such as dancing, a walking group and circuit training, to introduce participants to a range of options to suit them. We also chose to combine our detailed COM-B analysis with an inductive thematic analysis to explore wider social and cultural influences on behaviour to fully inform the intervention design (currently being prepared for publication). The BCW approach closely guides the choice of intervention functions and BCTS, while other approaches, such as intervention mapping, require more input from the researcher to consider suitable theories for each determinant of behaviour and the conditions necessary for a chosen method of change to be effective [13]. Process evaluation will be important to assess the effectiveness of the more prescriptive BCW approach, as used for this specific intervention.

The BCTs harnessing social opportunity, that we identified through our data, have been shown to be effective in other behavioural interventions for African and Caribbean communities. Collectivism and the importance of social interaction for those of AfC ancestry is well reported [25]. A lack of social support has been identified as a particular barrier to health behaviour change in African American communities [26–28]; the provision of a social support group, or inclusion of a partner or family member, as part of lifestyle interventions have been shown to be particularly effective [28, 29]. Use of social support related BCTs has been reported to enhance the individual’s perceived control and self-efficacy and US researchers have suggested mobilising social support may be a particularly ‘therapeutic and cost-effective public health strategy’ for these communities [28]. Social norms are recognised as a powerful influencer of health behaviour [30] and we postulate that fostering social interaction as part of our intervention will encourage behaviour change in our participants.

Health education research in African American communities suggests patients prefer to receive information verbally rather than in a written format [31–33]; this fits with the close association African cultures have with ‘orality’, which refers to the importance placed on knowledge gained and transmitted by word of mouth and indirect forms of communication such as body movements [25]. This supports our choice of BCTs such as demonstration (e.g. content delivered through games and physical activity classes), use of credible sources (e.g. use of videos to convey motivating messages from patients and respected health professionals) and social comparison (e.g. participants reporting their successes each week), which may be more helpful for UK AfC communities than use of written educational resources alone. Indeed, physical activity interventions that include demonstration of exercise in African American communities, have been shown to be more effective than those which simply advised increased activity [34].

Our analysis shows that a certain amount of skill and self-regulation is involved in self-managing diabetes well. This is reflected in the literature [35, 36]. The use of behaviour change techniques associated with control theory [37], such as goal setting, action planning and self-monitoring [22] in interventions to promote physical activity and healthy dietary change are supported by a number of systematic reviews [20–22]. These techniques may be useful to improve self-regulation, an important part of diabetes self-management [21], and to overcome physical opportunity barriers such as lack of time. Literature from studies in hypertensive African American women suggests that having these skills is associated with increased engagement in physical activity [38], however we are not aware of any behavioural change literature that cites the use of these behaviour change techniques in UK AfC communities.

Our finding that both African and Caribbean patients have difficulties in translating healthcare advice to cultural foods eaten at home agrees with a previous small study which just focused on patients of Caribbean descent [39]. This knowledge gap is likely to be a key factor in cultural accessibility to education and advice. While cultural identity was strongly associated with consumption of traditional foods in both ethnicities in our study it was particularly powerful for the Black African patients, who showed little Western dietary acculturation in their descriptions of their dietary preferences. Our data support NICE guidance [4] suggesting that culturally-relevant dietary advice needs to be central to any lifestyle intervention.

Our study used rigorous qualitative methods involving independent validation with an external qualitative research advisor, with participants and with a core AfC patient and community leader advisory team. Considerable effort was made to include groups not previously accessed, using community advocates to develop relationships and trust. In addition, stratification by gender and ethnicity enabled us to draw out factors relevant to different sectors of the community. Where possible we endeavoured to purposively sample to include a range of education attainment and socio-economic status, however, like most qualitative research there may be limitations on the generalisability of our results due to the self-selecting nature of this approach and the relatively small sample size. We recognise that the focus group facilitators being of White European ethnicity may influence the data collection and analysis and have addressed this where possible with active involvement of members of the communities in the analysis and interpretation of the findings.

## Conclusions

Through the use of COM-B and the BCW approach, we have recognised that people with T2D in our UK AfC communities are motivated to avoid long-term consequences of T2D and that having social support facilitates positive health behaviours. Walking and dancing are culturally relevant as is a desire to have a smaller waist. Our analysis has shown the importance of addressing motivational and social opportunity barriers to engaging in healthful diabetes self-management behaviour, as well as addressing gaps in knowledge. This study also highlights cultural barriers to lifestyle advice which may be generally relevant to healthcare practitioners supporting these communities. The BCW theoretical model successfully facilitated the explicit linkage of these identified theoretical behaviour change constructs with evidence-based behaviour change techniques. The resulting HEAL-D intervention is being evaluated in a randomised, controlled feasibility study with nested process evaluation, which will enable us to evaluate operationalisation of our chosen BCTs and their impact on behaviour change.

## Additional files

Additional file 1: Coding detail - Summary of coding tree for the qualitative data. (PDF 32 kb)

Additional file 2: Target behaviours - Summary of key potential behaviours and scoring. (PDF 56 kb)

Additional file 3: Topic guide - Overview of topic guide for focus groups. (PDF 82 kb)

Additional file 4: COREQ checklist – Completed checklist for this manuscript. (PDF 491 kb)

## Data Availability

The datasets used and/or analysed during the current study will be available from the corresponding author on reasonable request, following completion of the study and publication of associated PhD thesis.

## Abbreviations

AfC: African and Caribbean
APEASE: Acceptability, Practicability, Effectiveness/cost-effectiveness, Affordability, Safety/side-effects, Equity
BA: Black African
BC: Black Caribbean
BCT: Behaviour change technique
BCW: Behaviour change wheel
COM-B: Capability, Opportunity, Motivation and Behaviour
GP: General practitioner/Doctor
HEAL-D: Healthy Eating and Active Lifestyles for Diabetes
MRC: Medical Research Council
MVPA: Moderate to vigorous physical activity
SEM: Socio ecological model
SMART: Specific, Measurable, Achievable, Realistic, Time-based
T2D: Type 2 diabetes
TDF: Theoretical Domains Framework
TDF: Theoretical Domains Framework
