# Supplementary material for: Designing the Healthy Eating and Active Lifestyles for Diabetes (HEAL-D) self-management and support programme for UK African and Caribbean communities: a culturally tailored, complex intervention under-pinned by behaviour change theory
Source: BMC Public Health. 2019 Aug 20;19:1146. doi: 10.1186/s12889-019-7411-z (PMC6702734; doi:10.1186/s12889-019-7411-z)
Supplement: Supplementary file 1 — Coding detail - Summary of coding tree for the qualitative data. (PDF 32 kb) [file 12889_2019_7411_MOESM1_ESM.pdf]

Additional file 1: Description of coding

COM-B Domain	TDF	Themes coded under this node
Psychological CAPABILITY	Knowledge	Understanding of role of different foods, exercise & medication<br>Understanding of health guidance<br>Questions & gaps in understanding of self-management<br>Understanding of origins, causes and management of diabetes
	Memory attention & decision processes	Planning and organisation
	Behavioural regulation	Concentration, managing carb content<br>Avoiding temptation and determination<br>Organising self & self-management planning
Physical CAPABILITY	Skills	Stamina to exercise<br>Ability to count calories
Social OPPORTUNITY	Social influences	Influence of church communities<br>Cultural beliefs and values<br>Cultural norms<br>Friends & family influences<br>Positive & negative influences from others
Environmental OPPORTUNITY	Environmental context & resources	Access to medical care<br>Time constraints<br>Facilities in local community (e.g commercial weight management programmes, gyms, outside space, shopping facilities)<br>Cost & financial<br>Roles & working lives
Motivation REFLECTIVE	Beliefs about capabilities	Attitudes and feeling about diabetes<br>Fatalism and faith influences<br>Past successes & failures (weight management, regular exercise, dietary changes)
	Intentions, goals	Belief in the value of self-management behaviours<br>Desire to manage long term diabetes risk
	Beliefs about consequences	Understanding of the long-term risks of diabetes<br>Understanding of the effects and benefits of lifestyle changes

	Identity	Cultural identity<br>Role in family/community
Motivation AUTOMATIC	Reinforcement/ habit	Usual diet and lifestyle choices<br>Innate drivers e.g. hunger, cravings
	Emotions	Feelings about diabetes and complications
