# Supplementary material for: Designing the Healthy Eating and Active Lifestyles for Diabetes (HEAL-D) self-management and support programme for UK African and Caribbean communities: a culturally tailored, complex intervention under-pinned by behaviour change theory
Source: BMC Public Health. 2019 Aug 20;19:1146. doi: 10.1186/s12889-019-7411-z (PMC6702734; doi:10.1186/s12889-019-7411-z)
Supplement: Supplementary file 2 — Target behaviours - Summary of key potential behaviours and scoring. (PDF 56 kb) [file 12889_2019_7411_MOESM2_ESM.pdf]

Outcome	Behaviour	Impact	Liklihood	Splillover	Measurement	Score
Increase fibre	Eat wholegrains	++	+	++	+	6
Increase fibre	Choose wholegrains over refined foods and flours	++	++	++	+	7
Increase fibre	Add more vegetables to dishes and to plate	+++	++	+++	+	9
Increase MVPA to follow guidelines	Engage in cardiovascular exercise of choice for 30 mins/day 150 mins/week	++++	++	+++++	+++	15
Increase MVPA to follow guidelines	Count number of steps	+++	++++	+++++	+++	15
Increase MVPA to follow guidelines	Walk 10,000 steps (or graded increase)	++++	++++	+++++	+++	16
Increase MVPA to follow guidelines	Walk at M/V intensity for 30 mins/day or 150 mins/week	++++	++++	+++++	+++	16
Increase strength exercises	Do resistance training twice a week	+++	+	++	+	7
Increase strength exercises	Do a strength workout at a gym twice a week	++++	+	+++++	+	11
Increase strength exercises	Do self-devised strength routine at home	++++	+	+++++	+	11
Lose 5k or 5% BW	Do resistance training twice a week	+++	+	++	+	7
Lose 5k or 5% BW	Count calories to reduce intake	+++++	+	+++	++	11
Lose 5k or 5% BW	Engage in cardiovascular exercise of choice for 30 mins/day or 150 mins/week	+++++	++	++++	+++	15
Lose 5k or 5% BW	Self-montor weight against a target e.g. appropriate BMI	+++++	+	+++++	++	13
Lose 5k or 5% BW	Walk 10,000 steps (or graded increase)	++++	++++	+++++		13
Lose 5k or 5% BW	Self-monitor waist circumference	+++++	++++	+++++	++	16
Reduce calories	Don't eat because stressed or upset	+	+++	+	+	6
Reduce calories	Know when eating for emotional reasons	++	+	++	+	6
Reduce calories	Measure and rate hunger	++	+	++	+	6
Reduce calories	Choose wholegrains over refined foods and flours	++	++	++	+	7
Reduce calories	Cut fat off meat	+++	++	++	+	8
Reduce calories	Eat low fat diary options such as yoghurt and milk	+	++++	++	+	8
Reduce calories	Don't drink alcohol	++	+++++	+	+	9
Reduce calories	Stop eating when full	+++	++	+++	+	9
Reduce calories	Don't eat if not hungry	++++	+++	+++	+	11
Reduce calories	Measure and limit portion sizes (using hand portions)	+++++	++++	+++++	+	15
Reduce carb portion	Measure blood glucose after eating different foods* Not affordable	++++	+	++++	+	10
Reduce carb portion	Switch starchy carbohydrates for non-starchy vegetables	+++++	+	+++	+	10
Reduce carb portion	Weigh portions to manage CHO intake	+++++	+	+++	+	10
Reduce carb portion	Restrict portion size of CHO each meal to a fist or flat hand	+++++	++++	+++++	+	15
Reduce portion size	Measure portion sizes in g	++++	+	++++	+	10
Reduce portion size	Eat portions in line with the guidance given (g or hand sizes)	+++++	+++	+++++	+	14
Reduce portion size	Measure portion sizes in hand sizes	+++++	++++	+++++	+	15
Reduce saturated fat	Don't eat skin of chicken	++	++++	++	+	9
Reduce saturated fat	Use olive oil instead of coconut oil	+++	+++	++	+	9
Reduce saturated fat	Use reduced fat coconut milk/cream	+++	+++	++	+	9
Reduce saturated fat	Cut excess fat off meat	+++	++++	++	+	10
Reduce saturated fat	Measure coconut oil with spoon when cooking to limit quantity	+++	++++	++	+	10
Reduce saturated fat	Switch red meat for fish, poultry or beans	++++	+++	++	+	12
Reduce saturated fat	Switch saturated fat sources to unsaturated	+++++	+++	+++++	+	14
Restrict salt to recommended levels	Don't eat takeaway	+++	+	++	+	7
Restrict salt to recommended levels	Eat takeaway options that are not high in salt	++	+++	++	+	8
Restrict salt to recommended levels	Read salt labels on packaged foods	++	+++	++	+	8
Restrict salt to recommended levels	Use low salt seasonings	++++	++	+++	+	12
Restrict salt to recommended levels	Use spices and herbs instead of seasoning cubes	++++	++	+++	+	12
