# Supplementary material for: Designing the Healthy Eating and Active Lifestyles for Diabetes (HEAL-D) self-management and support programme for UK African and Caribbean communities: a culturally tailored, complex intervention under-pinned by behaviour change theory
Source: BMC Public Health. 2019 Aug 20;19:1146. doi: 10.1186/s12889-019-7411-z (PMC6702734; doi:10.1186/s12889-019-7411-z)
Supplement: Supplementary file 3 — Topic guide - Overview of topic guide for focus groups. (PDF 82 kb) [file 12889_2019_7411_MOESM3_ESM.pdf]

HEAL-D Patient Focus Group

CONSENT in advance.

INTRODUCE SELF

BACKGROUND TO PROJECT

RESEARCH BRIEF

- Participation is entirely **voluntary.** We will be giving you a £20 gift voucher at the end of today's session to thank you for your time but you are not in any way obliged to take part.
- participation in our project is **anonymous,** only the people in this room will know of your participation and we would ask that everybody here today respects the **confidentiality** of the information that is shared.
- We will be recording today's discussion on a tape to allow us to go back and listen to all the information you give us but we will not be sharing that with anybody outside of the research team and you will not be identified by name on any of our records.

HOUSE-KEEPING

- If you need to go to the toilet it is located…….
- Aim for this to be a discussion so join in the conversation at any time, but do try to speak one at a time so we can all join in the discussion.

FOCUS GROUP

Ice-breaker:

Please could you just briefly introduce yourself and tell us a bit about you

Main discussion: see question guide.

Close: 'so that brings our discussion to an end. I'd like to thank everybody very much for their participation today, the information you have shared is so valuable. I would like to reiterate what I said at the start that we would ask everybody to keep today's discussions confidential.

Travel expenses.

Gift voucher (£20).

Questionnaire.

QUESTION GUIDE

**People often talk about eating healthily when you have diabetes.**<br>**What does "eating healthily for diabetes" mean to you?**	
**<u>with images and foods onto the table</u>**<br><br>1. **Prompt :** <u>Can you each pick up an image or food that you think is either good to eat if you have diabetes or that you should limit if you have diabetes……</u><br><br>What dietary changes are recommended?<br>How do you choose what size portions to eat?<br>What changes have you made to your food and the way you cook?<br>What have you found difficult about changing the food you eat?<br><br>2. **Prompt:** <u>What makes it hard to make changes?</u><br><br>When you have friends and family to visit do you eat different foods?<br>How do foods differ when there's a celebration?<br>What foods are offered as part of religious events<br>Who does the shopping? Cooking?	
**Q2. ACTIVITY: Watch this short film. What physical activity do you do regularly?**	
**PROMPT 1. What stops you being more active?/What would help you be more active.**<br>*(probe time/facilities/cost/motivation/safety)*<br><br>**PROMPT 2. Which of these physical activities appeals to you most and why?**<br><br>**Show images of dancing/ gym/classes running/swimming.**	

Are they any other exercise activities you like to do?<br>What stops you getting the exercise you want?<br><br>**PROMPT 3. What advice have you been given about levels of physical activity?**<br>What are the guidelines?<br>Do you achieve these levels?<br>What physical activity do you do……………………………………….?<br>*(probe walking/gym/running/classes/exercise at home)*	
<u>Q3 LOOKING AT THESE PICTURES WHICH OF THESE WOMEN/MEN DO YOU THINK HAS A HEALTHY BODY SHAPE? (why? Why? Why?)</u>	
WAIT, WAIT, WAIT<br>1. <u>Could we discuss your experiences of trying to lose weight………………….?</u><br>*Can you tell me about that? (probe what worked/What was hard/How did the people close to you help/hinder?)*<br>How do you feel about losing weight?<br>How do you know what is the right weight? (probe scales)<br>What factors influence your weight?<br>How does your partner influence your views about weight?<br>What would help you lose weight?	
**<u>Q4. We are working to design a specific programme of advice and support for African & Caribbean patients we would like to ask your opinion on some specific issues?.......</u>**	
**<u>Healthy eating</u>**<br>What do you think would help people to make changes to their diet……..?<br>Practical help - Cooking classes? Recipes? Portion sizes?<br>What messages would motivate participants to change?	
**<u>Physical Activity</u>**<br>What activities could we offer to encourage people to be active?<br>What times?<br>What location?	

What factors should we consider? (sports shoes/clothes/safety/cultural issues/faith issues?	
**Weight**<br>How do we encourage people to lose weight?<br>What would people thing about being weighed? Do people have scales?	
**Practical arrangements (start with this)**<br>Time of day for sessions?<br>Where?<br>What equipment needs to be given?<br>Is child care needed<br>Social media & contact<br>Format of information?	
**Is there anything else we should consider?**	
**CLOSE**	
Thanks<br>Re-state confidentiality<br>Gift vouchers<br>Screening data	
